# Spatial pattern and predictors of malaria in Ethiopia: Application of auto logistics regression

**DOI:** 10.1371/journal.pone.0268186

**Published:** 2022-05-20

**Authors:** Yamral M. Warkaw, Aweke A. Mitku, Melkamu A. Zeru, Muluwerk Ayele

**Affiliations:** 1 Department of Statistics, College of Science, Bahir Dar University, Bahir Dar, Ethiopia; 2 Schools of Mathematics, Statistics and Computer Science, College of Agriculture Engineering and Science, University of KwaZulu-Natal, Durban, South Africa; University of Uyo, NIGERIA

## Abstract

**Introduction:**

Malaria is a severe health threat in the World, mainly in Africa. It is the major cause of health problems in which the risk of morbidity and mortality associated with malaria cases are characterized by spatial variations across the county. This study aimed to investigate the spatial patterns and predictors of malaria distribution in Ethiopia.

**Methods:**

A weighted sample of 15,239 individuals with rapid diagnosis test obtained from the Central Statistical Agency and Ethiopia malaria indicator survey of 2015. Global Moran’s I and Moran scatter plots were used in determining the distribution of malaria cases, whereas the local Moran’s I statistic was used in identifying exposed areas. The auto logistics spatial binary regression model was used to investigate the predictors of malaria.

**Results:**

The final auto logistics regression model was reported that male clients had a positive significant effect on malaria cases as compared to female clients [AOR = 2.401, 95% CI: (2.125–2.713) ]. The distribution of malaria across the regions was different. The highest incidence of malaria was found in Gambela [AOR = 52.55, 95%CI: (40.54–68.12)] followed by Beneshangul [AOR = 34.95, 95%CI: (27.159–44.963)]. Similarly, individuals in Amhara [AOR = 0.243, 95% CI:(0.195–0.303], Oromiya [AOR = 0.197, 955 CI: (0.158–0.244)], Dire Dawa [AOR = 0.064, 95%CI(0.049–0.082)], Addis Ababa[AOR = 0.057,95%CI:(0.044–0.075)], Somali[AOR = 0.077,95%CI:(0.059–0.097)], SNNPR[OR = 0.329, 95%CI: (0.261–0.413)] and Harari [AOR = 0.256, 95%CI:(0.201–0.325)] were less likely to had low incidence of malaria as compared with Tigray. Furthermore, for one meter increase in altitude, the odds of positive rapid diagnostic test (RDT) decreases by 1.6% [AOR = 0.984, 95% CI: (0.984–0.984)]. The use of a shared toilet facility was found as a protective factor for malaria in Ethiopia [AOR = 1.671, 95% CI: (1.504–1.854)]. The spatial autocorrelation variable changes the constant from AOR = 0.471 for logistic regression to AOR = 0.164 for auto logistics regression.

**Conclusions:**

This study found that the incidence of malaria in Ethiopia had a spatial pattern which is associated with socio-economic, demographic, and geographic risk factors. Spatial clustering of malaria cases had occurred in all regions, and the risk of clustering was different across the regions. The risk of malaria was found to be higher for those who live in soil floor-type houses as compared to those who lived in cement or ceramics floor type. Similarly, households with thatched, metal and thin, and other roof-type houses have a higher risk of malaria than ceramics tiles roof houses. Moreover, using a protected anti-mosquito net was reducing the risk of malaria incidence.

## Introduction

Malaria is transmitted to humans by five species of single-cell, eukaryotic Plasmodium parasites (mostly Plasmodium falciparum and Plasmodium vivax) through the bite of an infective female Anopheles mosquito. Malaria parasites proliferate and reproduce in people, first in the liver cells and then exponentially in the infected person’s red blood cells. The blood type of the parasite lifecycle causes the symptoms of malaria in humans when the parasites mature and leave the liver to infect red blood cells [1]. Malaria is the most public tropical disease and is still prevalent in tropical and subtropical regions, including parts of Africa, Asia and the Americas. It is one of the leading causes of illness and death in large parts of developing countries, mainly in Africa [2].

According to the world health organization report (WHO, 2020), an estimated 1.5 billion malaria cases and 7.6 million malaria death in the world. Perfect estimates of malaria distribution are required for planning, implementation and evaluation of malaria control programs. Hence, there is a need for precise estimates about the number of people at risk of malaria to optimize the use of limited resources in a high-risk area [3]. Malaria was the leading cause of outpatient visits, health facility admissions, and in-patient mortality in Ethiopia. According to the Federal Ministry of Health of 2009, 12% were outpatient visits and 9.9% were admissions. However, due to the lacks of access to health care, 36% of the population is likely to underestimate the true prevalence of malaria. Increasing our understanding of malaria distribution and its association with other diseases could lead to improvements in malaria control efforts [4].

In Ethiopia, rainfall and temperature are the most important determinants of malaria transmission, and the distribution is highly seasonal in many regions but may have a nearly constant transmission in some other areas; at the district level. Malaria outpatient caseloads may vary several-fold from year to year and season to season in unstable epidemic-prone transmission pattern. Peak malaria transmission occurs season between September and December in most Ethiopia, after the main summer season from June to August. Malaria is a serious health problem in Ethiopia, affecting the socio-economic and health status of the country at large.

Based on Ethiopia Malaria Indicator Survey of 2015 (EMIS-2015), nearly 60% of Ethiopia’s population lives in the malaria region, and 68% of the country’s population is at high risk of spreading malaria. Malaria is more closely related to altitude and season than rainfall. In general, the top of malaria spread follows the main summer season each year (July to September). But in many parts of the south and west, the rainy season starts earlier in April and May. Consequently, malaria transmission tends to be highly heterogeneous geospatially, both within and between years. Furthermore, malaria in Ethiopia is characterized by generalized epidemics that occur every five to eight years in which the greatest rampant occurs [5].

In this work, in the absence of covariate, global and local autocorrelation metrics were utilized to identify uni-variate spatial autocorrelation. After that, an auto logistics spatial regression model was estimated and a diagnosis test was run to see if the variables were adequately represented to reflect the spatial dependency of the dependent variable. When data for geographical factors is available, spatial auto logistics models describe malaria morbidity variance by geographical location better than non-spatial model models. The spatial model is used to measure neighboring effects and is used in a variety of research projects Odhiambo, Kalinda [6] highlighted that geographically situated data analysis is one of the statistician’s key concerns, and as a result, it is becoming increasingly significant in other disciplines of research. Tests of spatial autocorrelation tests are used to determine the level of clustering and to make statistical inferences [7].

Prophylaxis medications have been extremely beneficial and are widely used to monitor malaria transmission control, however they are no longer effective in many tropical places due to drug resistance by the parasite. Insecticide-treated nets (ITN) are being more widely touted as an effective way to reduce malaria incidence [8]. This is accomplished by determining the regional distribution of malaria. Malaria and disease heterogeneity spatial model models can be used to count geographic variation threats. Regional states, zones, districts and kebeles were the administrative divisions of Ethiopia’s federal government (kebeles). Regional states consider and understand the most important characteristics that influence malaria clustering in the study area in this study. The purpose of this research is to look into the spatial pattern and predictors of malaria in Ethiopia.

## Materials and methods

### Data

The data from the Ethiopian malaria indicator survey (EMIS-2015), the third comprehensive survey conducted as part of the national Ethiopian malaria indicator survey in 2015, was used in this investigation. This survey evaluated Ethiopia’s progress in scaling up malaria prevention and control interventions. From 555 enumeration areas selected in the first step, cross-sectional survey data from a secondary source retrieved from EMIS-2015 was employed with a two-stage cluster sampling process. Between September and December 2015, a poll was performed to assess the national malaria strategic plan.

During the survey, 15,960 individuals had RDTs. The study was population-based cross-sectional, including participants of all ages, and samples were chosen using a two-stage cluster probability sampling technique to select 555 enumeration locations from across Ethiopia’s malaria zones. A weighted sample size of 15,239 people was used in this investigation. This study included all household areas in the 555 EAs with altitudes of 2,000 and 2,000–2,499 meters, while households with laboratory malaria tested (had RDTs), no specific latitude and longitude (has no specific cluster number) individual in an EMIS 2015 documented data, and malaria (>2,500m ASL) were excluded.

Socio-demographic characteristics, insecticide-treated nets condition and availability, insecticide-treated nets, indoor residual spray, presence of stagnant water, outdoor stay at night, housing condition, and health information about malaria were extracted from EMIS-2015.

Binary data from EMIS, which was documented in 2015, was utilized in this investigation. A publication of the central statistical agency and EMIS, established in 2015, provided the shape file for each area and EAs of the country. In 2015, we also collected social and economic data from the county as potential risk factors for malaria occurrence. Observations that are recognized at geographic positions *R*_1_,*R*_2_,*R*_3_………*R*_11_ identify spatial data in R (regions) coordinates in the plane or polygons are in two, three, or more dimensions [9].

### Statistical models

The auto logistics spatial regression model and exploratory geographic data analysis (Moran’s I, and Local indicators of spatial autocorrelation, primarily Moran scatter plot) were utilized. Spatial autocorrelation measures and tests how clustered or dispersed points are in space in proportion to their attribute values using a metric known as the spatial autocorrelation coefficient. When spatial autocorrelation is minimal or absent, neighboring points in a distribution tend to have different properties. Moran’s I and Geary’s C statistics are the most generally used measurements for the proximity of locations and the similarity of their attributes. These statistics primarily challenge the assumption of spatial independence or randomness by measuring the strength of spatial autocorrelation among neighboring areal units [10].

One of the most extensively utilized models for modeling spatially linked binary data is the auto logistics spatial regression model. Many researches have shown that including auto covariate variables into the auto logistics regression is effective in modeling binary data with observed covariates. It’s a variant of the generalized logistic regression model with a spatial autocorrelation term in the form of Euclidean distance. In statistical analysis, it solves the problem of spatial autocorrelation effect [11].

We had restricted our attention to a constant auto regression coefficient (*r*_*ii*_ = *r*) for all geographic indexes that can express the conditional probability of the occurrence of malaria disease as:

$$\pi_{i}=\frac{\text{exp}\;\left(x_{i}^{'}\beta +rAutocov_{i}\right)}{1+\text{exp}\;\left(x_{i}^{'}\beta +rAutocov_{i}\right)}\;\text{Where}\;Auto\;cov_{i}=\frac{\sum_{j=1}^{ki}w_{ij}p_{j}}{\sum_{j=1}^{ki}w_{ij}}$$

$$W_{ij}=\left\{\begin{array}{c} 1\;\text{if}\;\text{centroid}\;\text{of}\;\text{j}\;\text{is}\;\text{one}\;\text{of}\;\text{the}\;\text{k}\;\text{nearest}\;\text{centroids}\;\text{to}\;\text{that}\;\text{of}\;\text{i}\\ 0\;\text{otherwise} \end{array}\right\}$$

Where, *π*_*i*_ denotes the probability of an event occurring for every region; *X*_*i*_ is independent variable, *Auto cov*_*i*_ is the auto covariate variable, *β and r* are the coefficient of explanatory variable and coefficient of fixed auto covariate variable in the equation, *i* is the index of geographical region (cluster) respectively.

Malaria incidences and geographic risk factors for malaria are often positively auto correlated. The values of two similar units in space tend to be more similar than would be predicted by chance. As a result, models that overlook spatial autocorrelation may be incorrect due to an overestimation of an environmental variable’s importance. Nonetheless, models that included the spatial autocorrelation effect were important to the response variable, resulting in accurate results in estimating the spatial distribution of malaria illnesses, improving model accuracy and adaptability. By adding any spatial autocorrelation between geographic units by incorporating an auto covariate variable acquired from the binary logistic regression model, the binary logistic regression model is modified to the auto logistic regression model [11].

The auto covariate variable can be calculated from the predicted probability of occurrence, which is estimated by the binary logistic regression model using this equation.


$$Auto\;cov_{i}=\frac{\sum_{j=1}^{ki}w_{ij}p_{j}}{\sum_{j=1}^{ki}w_{ij}},\;\text{where}$$

*Autocov*_*i*_ is the weighted average of the probability of the geographical units among a set *k*_*i*_ neighbours of the geographical unit i, *w*_*ij*_ the spatial weight between the geographic unit i and j is given by $w_{ij}=\frac{1}{h_{ij}}$, where *h*_*ij*_ is the Euclidean distance between the centroid of geographic unit i and j, P_j_ represent the predicted probability estimated by the binary logistics regression model.

To measure the relevance of over dispersion, we can look at the value of the chi-square statistic of the dispersion parameter in statistical comparisons between logistics and auto logistics regression. The preferred model for the data can then be determined using a likelihood ratio (LR) test [12]. A Boolean map of reality (the presence/absence) of malaria is compared to a probability map using relative operating characteristics (ROC). R Software was used to model and analyze key malaria transmission predictors using geographical point pattern data. The spatial autocorrelation and mapping were studied using ArcGIS. Using backward selection methods for the variable wealth index and education level to excluded from the study.

### Ethical statement

Ethical approval had been obtained from Bahir Dar University Ethical approval committee, Bahir Dar University, Ethiopia. In data collection, there was no written or verbal consent from participants because of the use of secondary data obtained from Ethiopian malaria indicator survey (EMIS-2015).

## Results

The study result showed that, among the weighed sample of 15,239 individuals, 2876 were positive RDTs results. From the total of 2876 malaria cases, 1951(67.84%) households were in rural. Out of 8362 female households, 510(17.73%) have malaria cases and among 6878 male households, 2366(15.53%) have malaria cases. Among malaria-positive cases, 1218(42.35%) were non-educated, 1015(35.29%) had primary level of education, 424(14.74%) had secondary level of education, and 219(7.61%) had higher education. Similarly, 1879(65.33%) malaria case households were used surface water, 907(31.54%) were used protected water, 32(1.11%) were used tanker water, and the remaining 58(2.02%) were had other sources of water (Table 1).

**Table 1:** Socio-demographic characteristics of malaria in Ethiopia.

Variables	Categories of variable	Malaria positive
Sex	Female	510(3.35%)
	Male	2366(15.53%)
Education status	No education	1218(7.99)
	Primary	1015(6.7)
	Secondary	424(2.8)
	Higher	219(1.44)
Residence	Urban	925(0.98)
	Rural	1951(17.6)
Source of water	surface water	1879(12.33)
	Protected	907(5.9)
	Tanker	32(0.2)
	Other	58(0.4)

**Fig 1:**
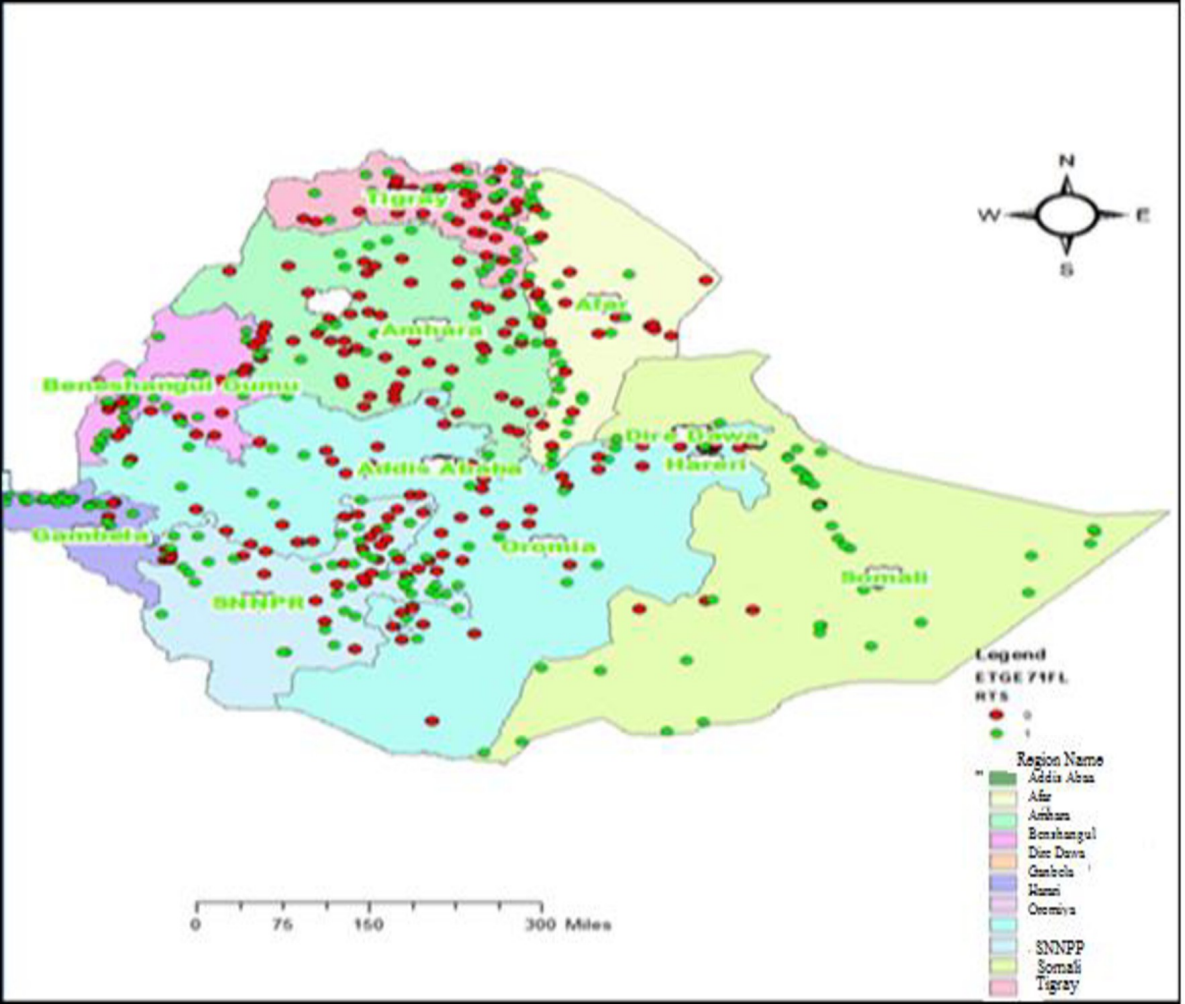
Spatial distribution of malaria test in Ethiopia, from EMIS 2015.

The spatial distribution of malaria in Ethiopia was presented in Fig 1. The red color indicates a negative malaria diagnosis test, and the green one indicates a positive malaria diagnosis test. Higher positive malaria cases were observed in the abuttal and northern parts of Ethiopia. Regionally, the highest malaria cases were observed in Somalia, Amhara, Oromiya, Tigray, Afar, SNNPR Beneshangul and Gambela followed by Afar and Harari. Low malaria cases were observed in the middle part of the country with the lowest records observed in Addis Ababa and Dire Dawa.

**Fig 2:**
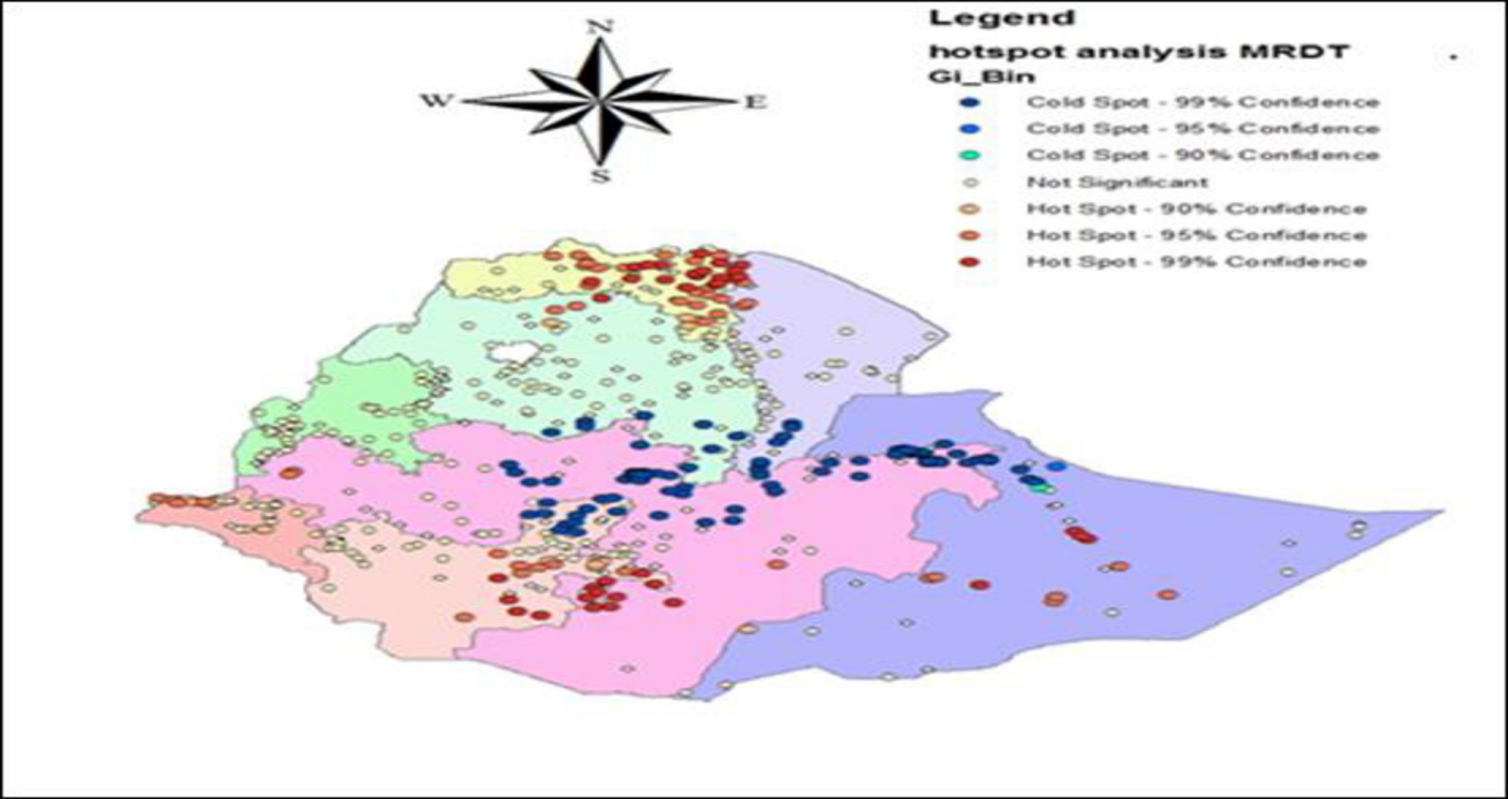
Hotspot analysis of malaria diagnosis test, from EMIS 2015.

In the Hot spot (Getis-Ord Gi*) analysis, the spatial hot spot analysis was predicted using incremental spatial autocorrelation maximum pick distance value 194.41 km and 237.986km (see S1 Fig). As shown in Fig 2, the red color is intense clustering of the high risk of malaria incidence (hot spot) in Ethiopia. The malaria rapid diagnosis test was clustered as high risk in Northern Amhara, Southwestern parts of Oromiya, Western parts of Gambela, Eastern and middle parts of Tigray, Western part of Afar, and central part of Somali regional states of Ethiopia. Whereas Addis Ababa, Dire Dawa, Southern parts of Amhara, Southern parts of Afar, Harari, Northern parts of Somali, Central Oromiya, and Northeastern part of Southern Nations, Nationalities, and people’s Regional states of Ethiopia were less risk area for malaria cases (Fig 2).

The geographical distribution of malaria cases on spatial clustering of malaria in Ethiopia based on the Local Moran’s I Statistic. The spatial pattern on the right side the critical value is greater than 2.58. It show that high rate of malaria occurred over study area. The critical value is the global autocorrelation show that there is spatial autocorrelation exists over the whole regions. As S2A Fig, shows Moran’s I of response variable code as 0 and 1 whereas S2B Fig, shows Moran’s I of response variable code as -1 and 1 both positive spatial autocorrelation indicates that region are located near to other region with similar values, either regions with high values on the variable being located near to regions also with high values or the opposite condition low values nearby other low values.

**Fig 3:**
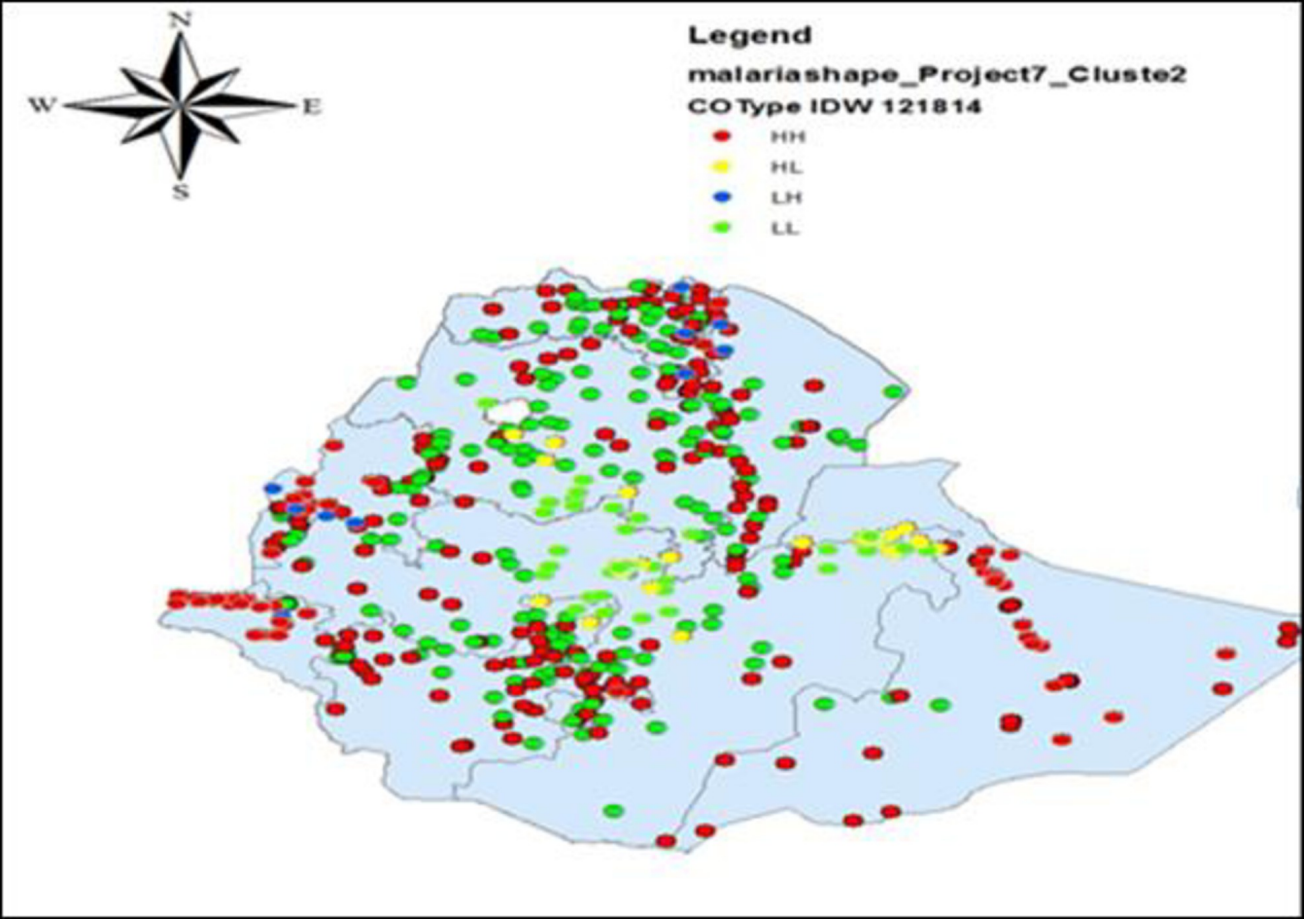
Spatial clustering of malaria in Ethiopia based on the Local Moran’s I statistic, from EMIS 2015.

As shown in Fig 3, the red color indicates high value surrounded by high value (HH), the green color indicates low value surrounded by low value (LL), the yellow color indicates high value surrounded by low value (HL) and the blue color shows low value surrounded by high value (LH). Therefore from the figure red and green colors are higher than the remaining two colors that indicate positive spatial autocorrelation. The hot spot regions were SNNP (all panels) and Oromiya, Beneshangul (middle and right), Harari, Somali, Gambella (south panels), north-west Amhara, and Central Tigray regions. The two town administrations (Addis Ababa and Dire Dawa), central Oromiya, Eastern Amhara, and Harari (left and right panels) regions were indicated as cold spot regions. The outliers were found on Addis Ababa and Dire Dawa (left), Oromiya, Harari, Amhara, Beneshangul-gumuz, SNNP, Afar (left and right), Gambella and Somali (left) regions. From Fig 3, the LISA of malaria was low value surrounded by low value in central parts of Amhara and Oromiya. Whereas, LISA of malaria was high value surrounded by high value in borders of all regions in Ethiopia. Each point on the map represents a single enumeration area with a number of malaria cases. The red (HH) color indicates malaria hotspot areas, the green (LL) color indicates malaria cold spot areas, the yellow (HL) and blue (LH) colors indicate outliers.

**Fig 4:**
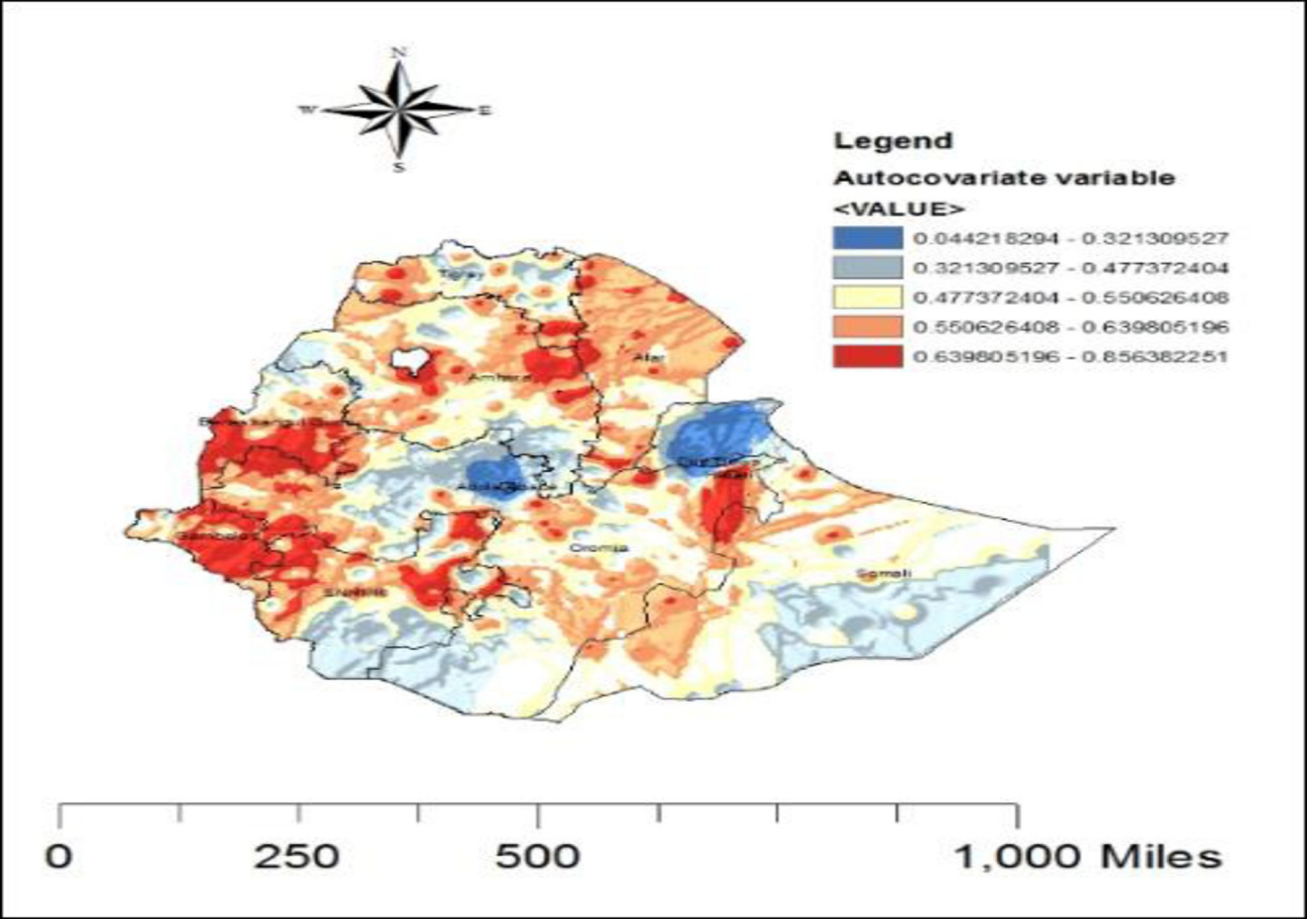
Predicted spatial effects from the malaria case in Ethiopia, from EMIS 2015.

Spatial autocorrelation (auto covariate) of malaria is almost predictable as human populations -live in spatial clusters rather than in random distributions of regions. As shown in Fig 4, the spatial distribution of the auto covariate variable represents the residual spatial autocorrelation term in the auto logistics regression model. The red colors showed Gambella, Beneshangul Gumuz, the common boundary of Afar and Amhara, the west part of Somalia and Oromiya in the south and east region were at high risk. Announcing the spatial auto covariate variable reflects data smoothing process, reducing local spatial dependence between geographical units to present the inherent spatial difference and tendency. The spatial auto covariate variable has the same unit of the malaria rapid diagnostics test, which also represents the probability of the malaria disease occurrence.

The final auto logistics regression model was reported that male clients had a positive significant effect on malaria cases as compared to female clients [AOR = 2.401, 95% CI: (2.125–2.713) ] which is above two times more likely to have malaria positive cases. The study result also revealed that the type of toilet facility had a significant effect on positive malaria tests. The larger estimated odds ratio for pit latrine toilet users [AOR = 3.14, 95% CI: (2.56–3.838)] and for bucket toilet facility users [AOR = 0.752, 95% CI: (0.6638–0.851)] shows that the probability of malaria positives test of bucket toilet facility user were less likely than flush toilet users. In contrast, clients who used pit latrine toilets were more likely to have positive malaria test than flush toilet users. The results also found Gambela[AOR = 52.548, 95%CI:(40.537–68.118)] and Beneshagul [AOR = 34.946, 95%CI:(27.159–44.963)] region of the country were related to the higher likelihood of malaria positive test results. Individuals who were in Amhara [AOR = 0.243, 95% CI:(0.195–0.303], Oromiya [AOR = 0.197, 955 CI:(0.158–0.244)],Dire Dawa [AOR = 0.064,95%CI(0.049-0-0.082)], Addis Ababa[AOR = 0.057, 95%CI(0.044–0.075)] Somali [AOR = 0.077,95%CI:(0.059–0.097)], SNNPR[AOR = 0.329, 95%CI: (0.261–0.413)] and Harari [OR = 0.256, 95%CI:(0.201–0.325)] are less likely to have malaria case as compared to individuals who were in Tigray. Based on the result of Table 2 the odds of having malaria decreased by 82.4% for individual with a one year increased age [AOR = 0.176, 95% CI: (0.1758–0.177)] holding other covariates constant.

**Table 2:** Parameter estimates for auto logistics regression model.

Parameter	AOR	95% CI for AOR	P-value
Intercept	0.164	(0.100, 0.267)	0.001
Altitude	0.984	(0.984, 0.984)	0.004
Spatial effect	3.841	(3.1200, 4.727)	0.001
Gender (Ref. = female)			
Male	2.401	(2.125, 2.713)	0.001
Age	0.176	(0.1758, 0.177)	0.001
Regions(Ref. = Tigray)			
Addis Ababa	0.057	(0.044, 0.075)	0.001
Afar	0.302	(0.229, 0.397)	0.016
Amhara	0.243	(0.195, 0.303)	0.002
Beneshangul	34.946	(27.159, 44.963)	0.001
Dire Dawa	0.064	(0.049, 0.082)	0.001
Gambela	52.548	(40.537, 68.118)	0.001
Hararie	0.256	(0.201, 0.325)	0.002
Oromiya	0.197	(0.158, 0.244)	0.001
SNNPR	0.329	(0.261, 0.413)	0.008
Somali	0.077	(0.059, 0.097)	0.026
Toilet facility (Ref. = flushed)			
Bucket toilet	0.752	(0.6638, 0.851)	0.001
No facility	1.481	(0.726, 3.018)	0.279
Pit latrine	3.14	(2.568, 3.838)	0.001
Time to get water	1.001	(1.0001, 1.001)	0.001
Drinking water(Ref. = protected)			
Other	0.285	(0.102, 0.789)	0.015
Surface water	1.207	(1.070, 1.361)	0.002
Tanker	1.375	(1.028, 1.839)	0.031
Material of room’s wall(Ref. = mud)			
Cement	0.991	(0.701, 1.401)	0.961
Other	1.211	(1.019, 1.438)	0.029
Wood	1.004	(0.847, 1.190	0.959
Mosquito net (Ref. = No net)			
Protected net	0.653	(0.591, 0.721)	0.001
Unprotected net	1.014	(0.871, 1.180)	0.85
Cooking(Ref. = wood and charcoal)			
Electricity	0.34	(0.291, 0.397)	0.001
Fuel	0.08	(0.05, 0.109)	0.001
Not cooking at home	3.99	(1.481, 10.753)	0.006
Toilet facility(Ref. = no)			
Yes	1.671	(1.504, 1.854)	0.001
Residence(Ref. = rural)			
Urban	0.717	(0.630, 0.814)	0.001
Material of room’s floor (Ref. = cement)			
Soil floor type	29.817	(23.206, 38.312)	0.001
Other	22.69	(16.966, 30.343)	0.001
Wood planks	17.619	(13.481, 23.028)	0.0001
Material of room’s roof (Ref. = ceramic)			
Metal and tin	6.521	(5.049, 8.421)	0.001
Other	7.299	(3.717, 14.329)	0.001
Thatch	5.674	(4.361, 7.382)	0.001

➢ Ref. = reference

➢ AOR = Adjusted Odds Ratio

Furthermore, for a one meter increase in altitude, the odds of positive RDT decreases by 1.6% [AOR = 0.984, 95% CI: (0.984–0.984)]. With reference to individuals who used protected water, malaria RDT was higher than individuals who used surface water [AOR = 1.207, 95% CI: (1.070–1.361)] followed by tanker [AOR = 1.375, 95% CI: (1.028–1.839)]. The results also showed individuals whose residence was urban were 0.717 times lower risk of being malaria positive test as compared to rural[AOR = 0.717,95% CI:(0.630–0.814)]. The probability of malaria positives test of metal [AOR = 6.521, 95% CI: (5.049–8.421)], thatched [AOR = 5.674, 95% CI:(4.361–7.382] and other [AOR = 7.299, 95%CI (3.717–14.329)] roof material types used is higher than cement or ceramic roofs. Furthermore, individuals who live in soil floor type [AOR = 29.817, 95%CI: (23.206–38.31)] are 29.8times more likely to have positive malaria cases as compared to individuals who lived in cement floor type. Similarly, other types of the floor are 22.69 times more likely to have malaria positive result [AOR = 22.69, 95%CI: (16.966–30.343)]. Likewise, households who live in types of rooms with wood walls were 1.2 times more likely to have been malaria positive than those households who lived with mud walls [AOR = 1.004, 95%CI: (0.847–1.190)].

After adjusting other covariates, households with protected mosquito bed nets have decreased the odds of having malaria by 34.7% than those in households without mosquito bed nets [AOR = 0.653, 95%CI: (0.591–0.721)] while households who used unprotected net were 1.014 times more likely to had malaria as compared to households who did not use net [AOR = 1.014, 95%CI:(0.871–1.180)].

Similar to Moran’s result, the spatial variable has a positive significant effect where districts with lower levels of patient status were usually surrounded by districts with lower levels of patient status and that districts with a higher incidence of malaria cases were usually surrounded by districts with a higher incidence of malaria. By introducing the spatial auto covariate variable, (γ = 1.35) when Euclidean distance in the meter was increase by one unit, that decreased spatial auto covariate variable but when increased spatial autocorrelation, the odds were 3.841 times more likely [AOR = 3.841,95%CI: (3.1200–4.727)] of positive malaria diagnostic test for individuals. In spatial auto covariate variables, the contribution of the constant is reduced significantly in the auto logistics model. The spatial autocorrelation variable changes the constant from AOR = 0.471 for logistic regression to AOR = 0.164 for auto logistics regression. However, the spatial auto covariate variable can be comprehended as the spatial inherent residual to reflect spatial effect in space data, which can reduce bias in health risk assessment. The spatial auto covariate variable helped to remove inherent residual errors from the binary logistic regression model (Table 2).

## Discussion

This study aimed to investigate the risk factors of malaria based on the EMIS 2015 data using an auto logistics spatial analysis approach. The results indicate that both global and local spatial clustering of malaria incidence among the region was different which helps to the allocation of resources for prevention based on the rate of exposure.

From our analysis, the spatial effect had a positive significant effect on malaria cases, where districts with lower levels of patient status were usually surrounded by districts with lower levels of patient status, and that districts with a higher incidence of malaria cases were usually surrounded by districts with a higher incidence of malaria. The finding of this was also supported by the study finding of Omukunda, Githeko [13]. A result in this study was reported that malaria incidence varies according to gender and age with significant malaria incidence. It was also observed that local clustering of malaria incidence between pairs of regions within distance lags was significant. Furthermore, malaria hot spots were displayed as risk maps that are useful for monitoring and spatial targeting of prevention and control measures. This finding is consistent with the finding of Yeshiwondim, Gopal [14].

Moreover, the malaria rapid test and altitude had an inverse relationship. The study found that as altitude increased, the risk of malaria reduced. This could be because the high altitude environment is unsuitable for Anopheles mosquito breeding due to its steep structure, which prevents water from being collected after the rainy season. This result is consistent with the findings of the study and [15] conducted in Ethiopia and Ugwu and Zewotir [16] which took place in Nigeria. Malaria was found to be distributed differently throughout Ethiopian regions in this study. Because the specific characteristics of socio-economics, demography and their locations effect contact between humans and vectors, the study’s findings reveal that the level of malaria risk or case incidence varies widely throughout regions.

This finding is in line with the result of Ayele, Zewotir [17] and Baidjoe, Stevenson [18] while contradicting the finding of Aychiluhm, Gelaye [19] and Ayele, Zewotir [17]. Different study times, as well as sample size differences, could be the cause of the result’s inconsistency. According to this research, the current state of rooms is commonly described as a malaria sickness caused by poverty and low socioeconomic circumstances. Poor people are disproportionately affected by malaria transmission because they cannot afford mosquito nets, cement walls, cement floors, or metal roofs. Because poverty is linked to socioeconomic variables, it’s critical to comprehend the connections between malaria transmissions.

Furthermore, the source of water has been found as a productive factor against malaria cases among peoples in Ethiopia. It revealed that those who used surface water had a 20.7% increased risk of malaria positivity as compared to those who did use protected water and 37.5% increased risk of malaria among individuals who used tanker water as compared to protected water users. This result was contradicted with the study conducted by Aychiluhm, Gelaye [19] on determinants of malaria among under-five children in Ethiopia which stated that those who used unprotected water were 1.07% times less likely to be infected. The possible reason for the contradiction is due to the difference in the study population, this study was based on all aged group individuals while Aychiluhm et al. was based on under-five children.

Based on our study, among respondents who mostly use protected mosquito nets and unprotected mosquito bed nets, the odds of having malaria was decreased by 35% and 1.7% respectively as compared to those who did use mosquito nets. This study result is similar to the study result conducted by [20].

The spatial auto logistic regression result reflecting that the transmission of malaria infection by the mosquitoes over space and the effects of socio-economic demographic and geographic variable types of toilet use and place of residence are highly associated with transmission of malaria that determines the survival of mosquito over large areas which is consistent with [21] finding. In a similar case, the main material of the room’s wall, the main material of the room’s roof, the main material of the room’s floor, and the use of mosquito nets were found malaria risk factors which are similar to the study by Ayele, Zewotir [17]. Furthermore, the finding of this study was confirmed that the transmission of malaria in the study area is significantly clustered indicating high levels in the SNNP, Tigray, Somali, Gambela, Oromia, and low levels Addis Ababa, Harari, Dire Dawa. In other words, it is cogently dissimilar in the Amhara and Beneshangul Gummz region of Ethiopia. This result is also agreed with the study of [20].

The limitation of this study is that data was on a secondary source and the survey used a cross-sectional design to collect data as such no pivotal extrapolations can be made between malaria infection and its determinants and also not including seasonal variation. Despite these limitations, the study used survey data collected from a nationally representative sample the laboratory investigation for individual malaria rapid diagnosis test results had no latitude and longitude rather than enumeration area.

## Conclusions

This study found that the incidence of malaria in Ethiopia displays a spatial pattern which is dependent on socio-economic, demographic, and geographic risk variables. Significant local clustering of malaria transmission occurs among regions and within neighboring regions. Our study indicates that socio-economic, demographic, and geographic factors are responsible for the transmission of malaria disease. Additionally, malaria prevalence is low for male households than a female household and elders are at a lower risk.

Spatial clustering of malaria cases has occurred in all regions, and the risk of clustering was different across the regions. Therefore, this study result of spatial clustering of malaria in Ethiopia can be used in planning and implementation of malaria control strategies at a macro-geographic scale.

The risk of malaria was found to be higher for those who live in soil floor-type houses as compared to those who lived in cement or ceramics floor type. Similarly, individuals who live in thatched, metal and thin, and other roof-type houses have a higher risk of malaria than ceramics tiles roof houses. Moreover, using a protected anti-mosquito net was reducing the risk of malaria incidence.

## Supporting information

S1 Fig Spatial autocorrelation by distance. (TIF)

S2 Fig Spatial pattern of malaria in Ethiopia. (TIF)
