# Supplementary figures and images for: Spatial pattern and predictors of malaria in Ethiopia: Application of auto logistics regression
Source: PLoS One. 2022 May 20;17(5):e0268186. doi: 10.1371/journal.pone.0268186 (PMC9122179; doi:10.1371/journal.pone.0268186)

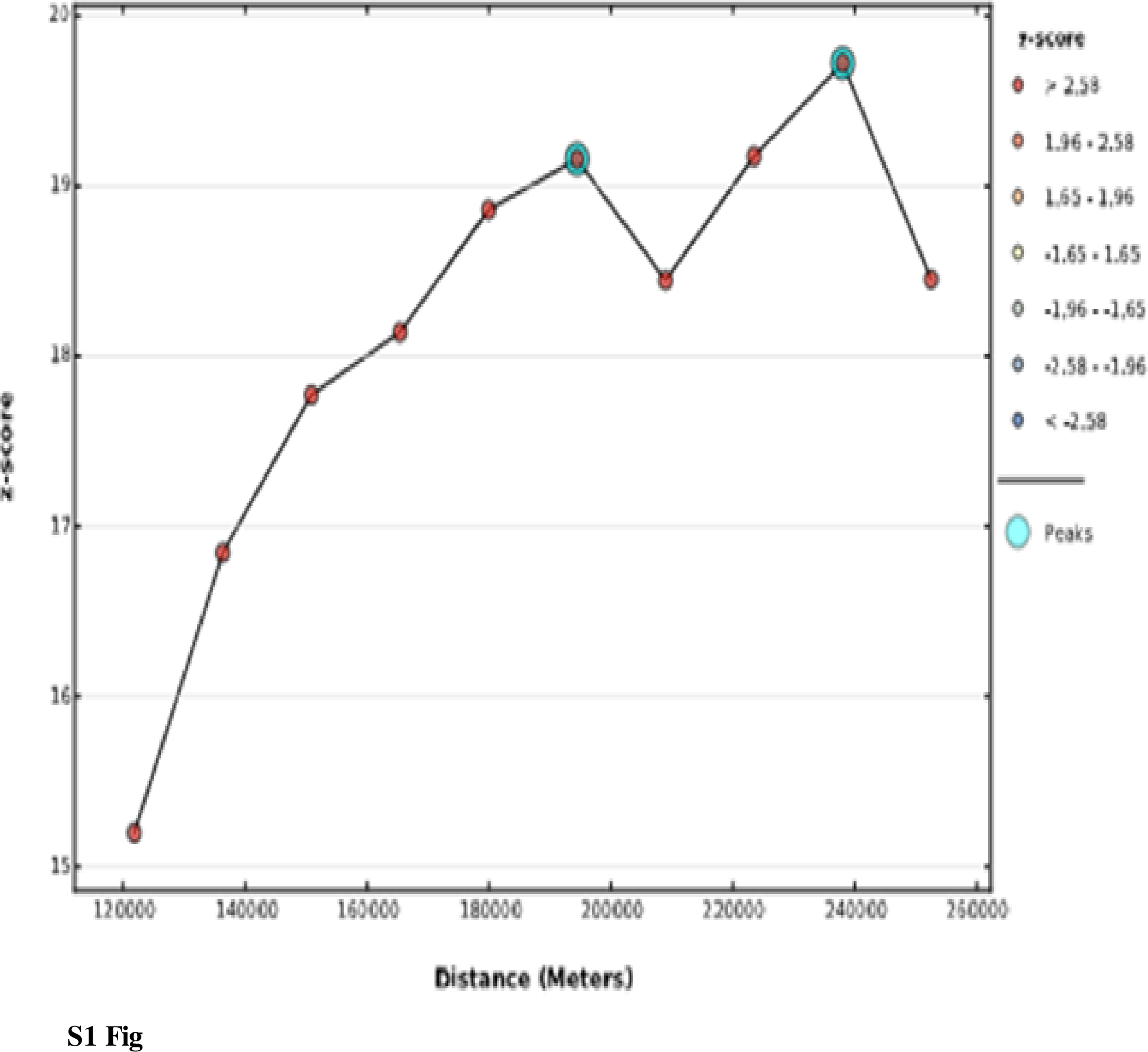

Supplement: S1 Fig — (TIF) [file pone.0268186.s001.tif]

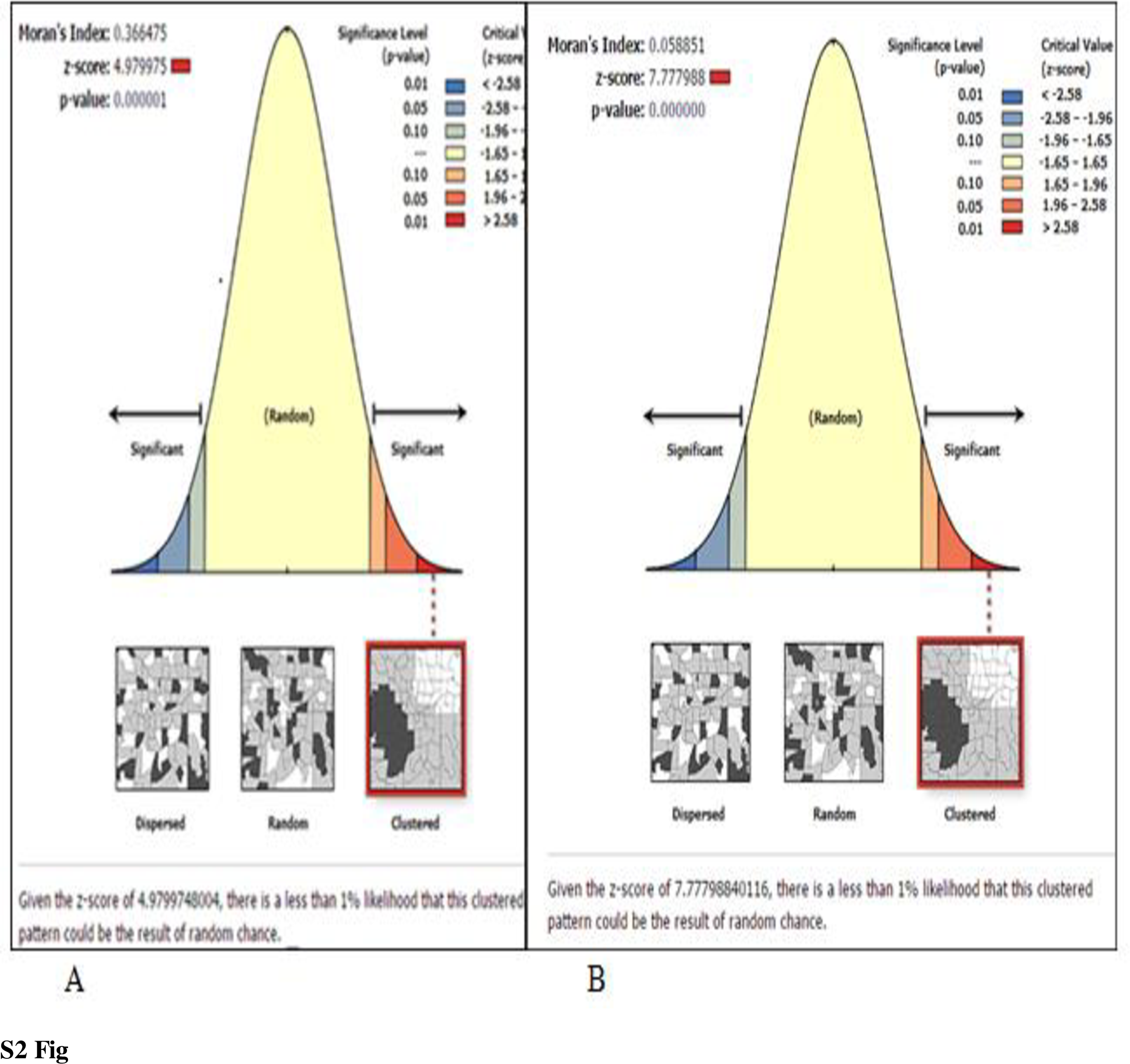

Supplement: S2 Fig — (TIF) [file pone.0268186.s002.tif]
